# Supplementary material for: Shark teeth zinc isotope values document intrapopulation foraging differences related to ontogeny and sex
Source: Commun Biol. 2023 Jul 11;6:711. doi: 10.1038/s42003-023-05085-6 (PMC10336102; doi:10.1038/s42003-023-05085-6)
Supplement: Supplementary file 2 — Description of Additional Supplementary Files [file 42003_2023_5085_MOESM2_ESM.docx]

**Description of Additional Supplementary Files**

**File name:** Supplementary Data 1

**Description:** Stable isotope results for all Carcharias taurus individuals analysed in this study together with information on their sex, as well as fork length and total length in cm.
