# Supplementary material for: Shark teeth zinc isotope values document intrapopulation foraging differences related to ontogeny and sex
Source: Commun Biol. 2023 Jul 11;6:711. doi: 10.1038/s42003-023-05085-6 (PMC10336102; doi:10.1038/s42003-023-05085-6)
Supplement: Supplementary file 4 — Reporting Summary [file 42003_2023_5085_MOESM4_ESM.pdf]

nature portfolio

Corresponding author(s): Jeremy McCormack

Last updated by author(s): Jun 25, 2023

Reporting Summary

Nature Portfolio wishes to improve the reproducibility of the work that we publish. This form provides structure for consistency and transparency in reporting. For further information on Nature Portfolio policies, see our Editorial Policies and the Editorial Policy Checklist.

Statistics

For all statistical analyses, confirm that the following items are present in the figure legend, table legend, main text, or Methods section.

n/a	Confirmed	
☐	☒	The exact sample size (*n*) for each experimental group/condition, given as a discrete number and unit of measurement
☐	☒	A statement on whether measurements were taken from distinct samples or whether the same sample was measured repeatedly
☐	☒	The statistical test(s) used AND whether they are one- or two-sided<br>*Only common tests should be described solely by name; describe more complex techniques in the Methods section.*
☐	☒	A description of all covariates tested
☐	☒	A description of any assumptions or corrections, such as tests of normality and adjustment for multiple comparisons
☐	☒	A full description of the statistical parameters including central tendency (e.g. means) or other basic estimates (e.g. regression coefficient) AND variation (e.g. standard deviation) or associated estimates of uncertainty (e.g. confidence intervals)
☐	☒	For null hypothesis testing, the test statistic (e.g. *F*, *t*, *r*) with confidence intervals, effect sizes, degrees of freedom and *P* value noted<br>*Give P values as exact values whenever suitable.*
☐	☒	For Bayesian analysis, information on the choice of priors and Markov chain Monte Carlo settings
☒	☐	For hierarchical and complex designs, identification of the appropriate level for tests and full reporting of outcomes
☒	☐	Estimates of effect sizes (e.g. Cohen's *d*, Pearson's *r*), indicating how they were calculated

Our web collection on statistics for biologists contains articles on many of the points above.

Software and code

Policy information about availability of computer code

Data collection	Thermo Scientific Mass Spectrometry Software.
Data analysis	All statistical analyses were conducted using the free program R software

For manuscripts utilizing custom algorithms or software that are central to the research but not yet described in published literature, software must be made available to editors and reviewers. We strongly encourage code deposition in a community repository (e.g. GitHub). See the Nature Portfolio guidelines for submitting code & software for further information.

Data

Policy information about availability of data

All manuscripts must include a data availability statement. This statement should provide the following information, where applicable:

- Accession codes, unique identifiers, or web links for publicly available datasets
- A description of any restrictions on data availability
- For clinical datasets or third party data, please ensure that the statement adheres to our policy

All data generated during this study are included in this published article (and its supplementary information files).

Human research participants

Policy information about studies involving human research participants and Sex and Gender in Research.

Reporting on sex and gender	*Use the terms sex (biological attribute) and gender (shaped by social and cultural circumstances) carefully in order to avoid confusing both terms. Indicate if findings apply to only one sex or gender; describe whether sex and gender were considered in study design whether sex and/or gender was determined based on self-reporting or assigned and methods used. Provide in the source data disaggregated sex and gender data where this information has been collected, and consent has been obtained for sharing of individual-level data; provide overall numbers in this Reporting Summary. Please state if this information has not been collected. Report sex- and gender-based analyses where performed, justify reasons for lack of sex- and gender-based analysis.*
Population characteristics	*Describe the covariate-relevant population characteristics of the human research participants (e.g. age, genotypic information, past and current diagnosis and treatment categories). If you filled out the behavioural & social sciences study design questions and have nothing to add here, write "See above."*
Recruitment	*Describe how participants were recruited. Outline any potential self-selection bias or other biases that may be present and how these are likely to impact results.*
Ethics oversight	*Identify the organization(s) that approved the study protocol.*

Note that full information on the approval of the study protocol must also be provided in the manuscript.

Field-specific reporting

Please select the one below that is the best fit for your research. If you are not sure, read the appropriate sections before making your selection.

☐ Life sciences ☐ Behavioural & social sciences ☒ Ecological, evolutionary & environmental sciences

For a reference copy of the document with all sections, see nature.com/documents/nr-reporting-summary-flat.pdf

Ecological, evolutionary & environmental sciences study design

All studies must disclose on these points even when the disclosure is negative.

Study description	Here we analyse zinc, carbon and nitrogen isotopes in sand tiger shark teeth to investigate dietary differences, related to sex and size, within this North Atlantic population.
Research sample	For this study, we take advantage of sand tiger shark teeth collected in the Delaware Bay between August 15 and 30 in 2012 as part of a joint tagging program between the University of Delaware and Delaware State University (see Haulsee et al. 2015, Haulsee et al. 2018). Approval for the joint tagging program, which occurred between 2007-2015, during which the teeth were originally collected comes from the Delaware Department of Natural Resources and Environmental Control (DNREC; 2012-021F), Delaware State University Institutional Animal Care and Use Committee (IACUC) and the University of Delaware IACUC (1259-2014-0). The teeth were originally collected for other isotope analytical purposes and for comparisons with fossilized teeth records (Kim et al. 2014). However, an advantage of this material, and why we selected it for this study, is that all teeth were collected within a short time span and information on sex and size of the individual is available.
Sampling strategy	We sampled teeth to represent sharks of varying sizes, i.e., juveniles and adults, as well as both sexes to investigate ontogenetic and sexual differences in diet.
Data collection	Mass spectrometry: Jeremy McCormack, Sora L. Kim, Molly Karnes
Timing and spatial scale	The analyses are not time dependent. Samples were collected between August 15 - 30, 2012 and within a 10 sqkm area within the Delaware Bay, Delaware, USA.
Data exclusions	All data are reported.
Reproducibility	All isotopic measurements included the analyses of internationally recognised standards and sample replicate analyses.
Randomization	Does not apply to this type of study.
Blinding	Does not apply to this type of study.

Did the study involve field work? ☒ Yes ☐ No

Field work, collection and transport

Field conditions	Sharks were caught and teeth collected in the Delaware Bay between August 15 and 30 in 2012 from a boat. The sea-state and weather were not recorded, but generally sampling occurred when wave heights were less than 1 m and when wind was less than 10 knots, regardless of precipitation.
Location	39N, 75W, Delaware Bay, Delaware, USA. Water depth: 10-30m
Access & import/export	Sharks were captured and released, and samples were collected under the authority of Delaware Department of Natural Resources and Environmental Control (DNREC; 2012-021F), Delaware State University Institutional Animal Care and Use Committee (IACUC) and the University of Delaware IACUC (1259-2014-0).
Disturbance	N/A

Reporting for specific materials, systems and methods

We require information from authors about some types of materials, experimental systems and methods used in many studies. Here, indicate whether each material, system or method listed is relevant to your study. If you are not sure if a list item applies to your research, read the appropriate section before selecting a response.

Materials & experimental systems

n/a	Involved in the study
☒	☐ Antibodies
☒	☐ Eukaryotic cell lines
☒	☐ Palaeontology and archaeology
☐	☒ Animals and other organisms
☒	☐ Clinical data
☒	☐ Dual use research of concern

Methods

n/a	Involved in the study
☒	☐ ChIP-seq
☒	☐ Flow cytometry
☒	☐ MRI-based neuroimaging

Animals and other research organisms

Policy information about studies involving animals; ARRIVE guidelines recommended for reporting animal research, and Sex and Gender in Research

Laboratory animals	The study did not involve laboratory animals
Wild animals	Sand Tigers were captured between using bottom longline techniques adapted from McCandless et al. (2007). Mainlines were approximately 305m of 0.64cm braided nylon, with barbless Mustad 12/0 circle hooks placed approximately every 12-13m. Each hook was baited with half of an Atlantic Menhaden (Brevoortia tyrannus). Sex, fork length (FL), total length (TL) and general condition were recorded for every Sand Tiger captured. After opportunistic teeth sample collection, and tagging following approved protocols (under the authority of Delaware Department of Natural Resources and Environmental Control (DNREC; 2012-021F), Delaware State University Institutional Animal Care and Use Committee (IACUC) and the University of Delaware IACUC (1259-2014-0), all sharks were released at the location of capture (within a 10 sqkm radius of sampling area within the Delaware Bay).
Reporting on sex	Analyses were sex-based by design. Sex of sharks was determined by the presence or absence of external claspers. We analysed teeth from 22 females (18 juvenile and 4 mature) and 32 males (19 juvenile and 13 mature).
Field-collected samples	The study did not involve laboratory work on wild-caught animals.
Ethics oversight	Samples were collected under the authority of Delaware Department of Natural Resources and Environmental Control (DNREC; 2012-021F), Delaware State University Institutional Animal Care and Use Committee (IACUC) and the University of Delaware IACUC (1259-2014-0).

Note that full information on the approval of the study protocol must also be provided in the manuscript.
